# A new species of *Viola* (Violaceae) from Guangdong Province, China

**DOI:** 10.3897/phytokeys.176.65443

**Published:** 2021-04-16

**Authors:** Yan-Shuang Huang, Ning Kang, Xiang-Jing Zhong, Wen-Bo Liao, Qiang Fan

**Affiliations:** 1 State Key Laboratory of Biocontrol and Guangdong Provincial Key Laboratory of Plant Resources, School of Life Sciences, Sun Yat-sen University, Guangzhou 510275, China Sun Yat-sen University Guangzhou China; 2 Management Bureau of Guangdong Xiangtoushan National Nature Reserve, Huizhou 516003, China Guangdong Xiangtoushan National Nature Reserve Huizhou China

**Keywords:** Morphology, new species, phylogeny, section *Diffusae*, *Viola huizhouensis*

## Abstract

*Viola huizhouensis* (Violaceae), a new species from Xiangtoushan National Nature Reserve of Guangdong Province in China, is described and illustrated. The new species is most similar to *V. guangzhouensis*, but it can be easily distinguished by its much stouter rhizome, lack of aerial stem, dense pubescence of the basal pedicel and the whole plant. Our phylogenetic analysis, based on ITS sequences, confirms that the new species belongs to V. sect. Diffusae.

## Introduction

Mount Xiangtoushan in Guangdong Province, China, is located in a subtropical zone with abundant rainfall, mainly a low-lying hilly landform, but has an extremely high altitude compared with the surrounding environment. A large area of granite in this region is exposed on the surface due to intense erosion and denudation and there are climax lithophytic vegetation communities with a high level of biodiversity. During fieldwork in March 2018 for the investigation of the biodiversity patterns in this mountainous region, a distinct new species, *Viola huizhouensis*, was collected on Mount Xiangtoushan.

*Viola* L. is the largest genus of family Violaceae, with approximately 525–600 species around the world (Ballard et al. 1998; Clausen 1929). This genus has a high level of morphological differentiation and there are hybridisation and horizontal evolution amongst sections and species (Marcussen et al. 2015). There are about 93–108 native *Viola* species in China which belong to four subgenera according to Yuzepchuk and Klokov’s (1949) classification, i.e. *Melanium*, *Chamaemelanium*, *Dischidium* and *Viola*. Amongst them, *Viola* is the largest subgenus, which includes nine sections and 78–95 species in China (Wang 1991; Chen et al. 2007).

## Material and methods

Leaf material of the putative new species and its related species *V. guangzhouensis*, was collected and stored with silica gel in zip-lock plastic bags until use for comparisons and taxonomical treatment. Specimens of *V. huizhouensis* and *V. guangzhouensis* were collected respectively from Darenyan, Xiangtoushan National Nature Reserve and Shaoshangling, Liuxi River State Forest Park in March 2018. Voucher specimens were deposited in the Herbarium of Sun Yat-sen University (**SYS**).

Total DNA was extracted with the modified CTAB method (Doyle and Doyle 1987). The regions of partial internal transcribed spacer 1, 5.8S ribosomal RNA gene and partial internal transcribed spacer 2 were amplified using previously-reported primers ITS1, ITS4 (White et al. 1990). PCR amplifications were performed following Fan et al. (2015). The sequences of the species and related ones, downloaded from NCBI, were aligned using MEGA 6.0 (Tamura et al. 2013) with ClusterW and subsequently manually adjusted. Phylogenetic constructions were carried out with Maximum Likelihood (ML). ML was run by Iqtree 2.0.3 (Minh 2020), selecting best-fit model TIM+F+G4 with 2000 bootstraps. Phylogenetic analyses, based on Bayesian Inference (BI) were carried out using MrBayes version 3.1.2 (Huelsenbeck and Ronquist 2001). Bayesian analysis was run with four chains for 200,000 generations with the first 25% of sampled trees discarded as burn-in. Main quantitative characteristics of the putative new species and *V. guangzhouensis* were statistically analysed using IBM SPSS version 22.0.

## Result

**Figure 1.**
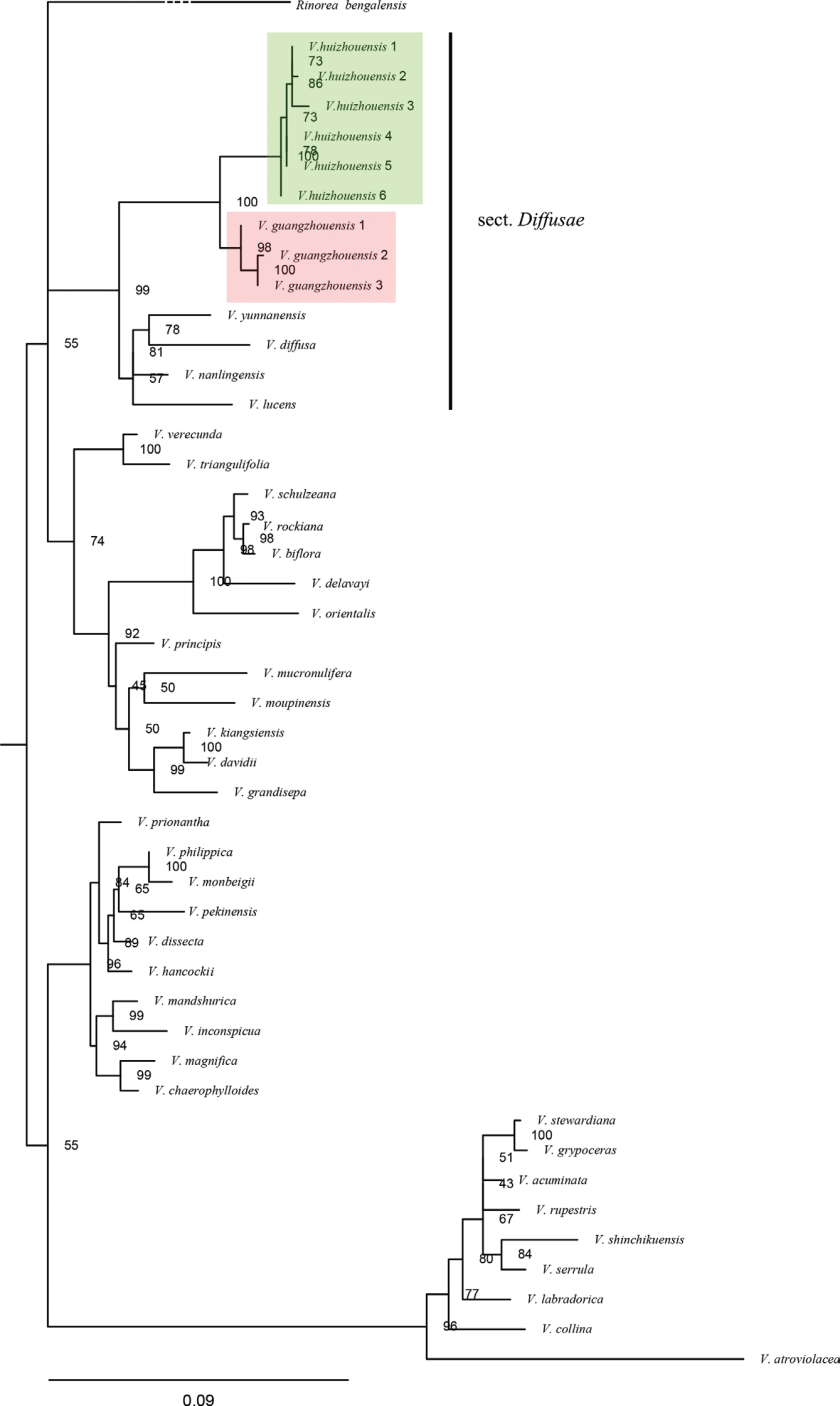
Maximum Likelihood tree of the new species and related species, Numbers beside branch nodes are bootstraps. Outgroups: *Rinorea bengalensis*. *Viola huizhouensis* clade is marked in green and *V. guangzhouensis* clade is marked in red.

The aligned length of ITS sequences was 638 bps in total. ML and BI analyses produced similar topology (Fig. 1 and Suppl. material 1: Fig. S1). The samples of the putative new species (*Viola huizhouensis*) and *V. guangzhouensis* A.Q. Dong, J.S. Zhou & F.W. Xing, clustered into their own species clade respectively, with strong support (BS = 100% for *V. huizhouensis* and BS = 98% for *V. guangzhouensis*). The *V. huizhouensis* clade clustered with the *V. guangzhouensis* clade, forming a sister relationship (BS = 100%). Then, the two species, together with *V. yunnanensis* W. Beck. & H. De Boiss., *V. diffusa* Ging., *V. nanlingensis* J.S. Zhou & F.W. Xing and *V. lucens* W. Beck., constituted a monophyletic clade (BS = 99%). Quantitative characteristics and a statistical analysis showed that there was a significant difference between *V. huizhouensis* and *V. guangzhouensis* in the diameter of rhizome and the leaf shape (Table 2).

### Viola huizhouensis

Taxon classificationPlantaeMalpighialesViolaceae

Y. S. Huang & Q. Fan sp. nov.

B4804353-9396-5B68-A508-D8A416C37BE6

urn:lsid:ipni.org:names:77216568-1

#### Type.

**Figure 2.**
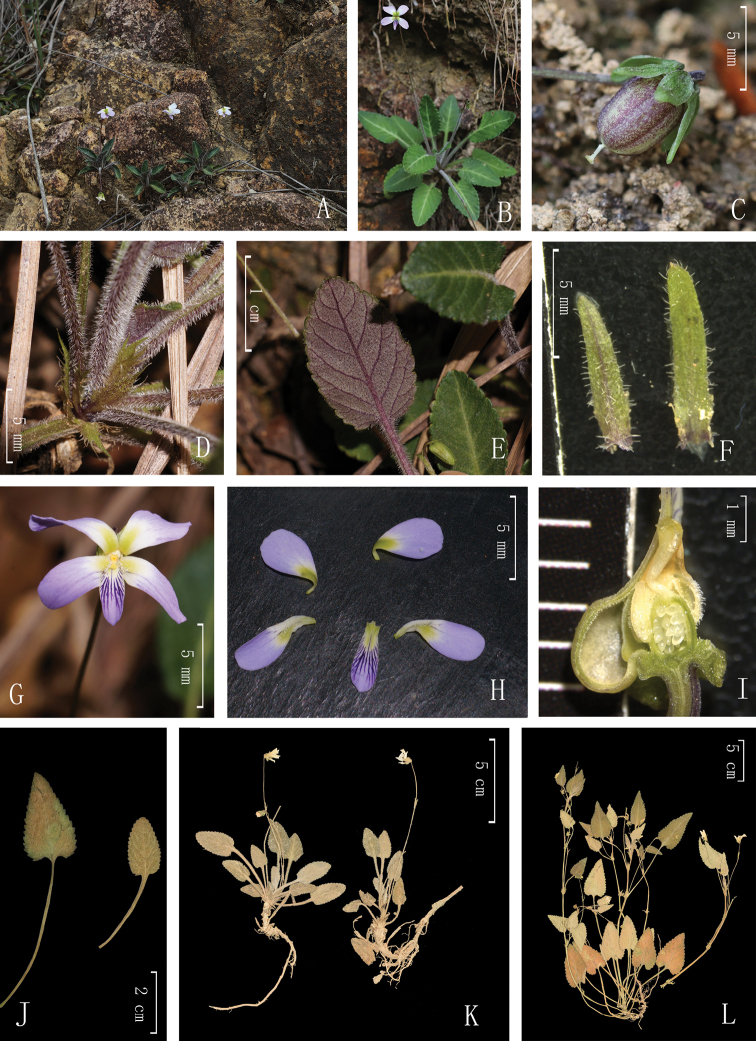
*Viola huizhouensis***A** habitat **B** habit **C** young capsule with sepals and bracteoles **D** stem with stipules **E** abaxial surface of leaf blade **F** bracteoles **G** flowers **H** petals **I** longitudinal section of stigma and ovary **J** comparison of leaf blades. Left, *V. guangzhouensis*. Right, *V. huizhouensis*. K. Specimen of *V. huizhouensis* (*Y.S. Huang and Q. Fan 1803*). L. Specimen of *V. guangzhouensis* (*Y.S. Huang 1804*).

**Figure 3.**
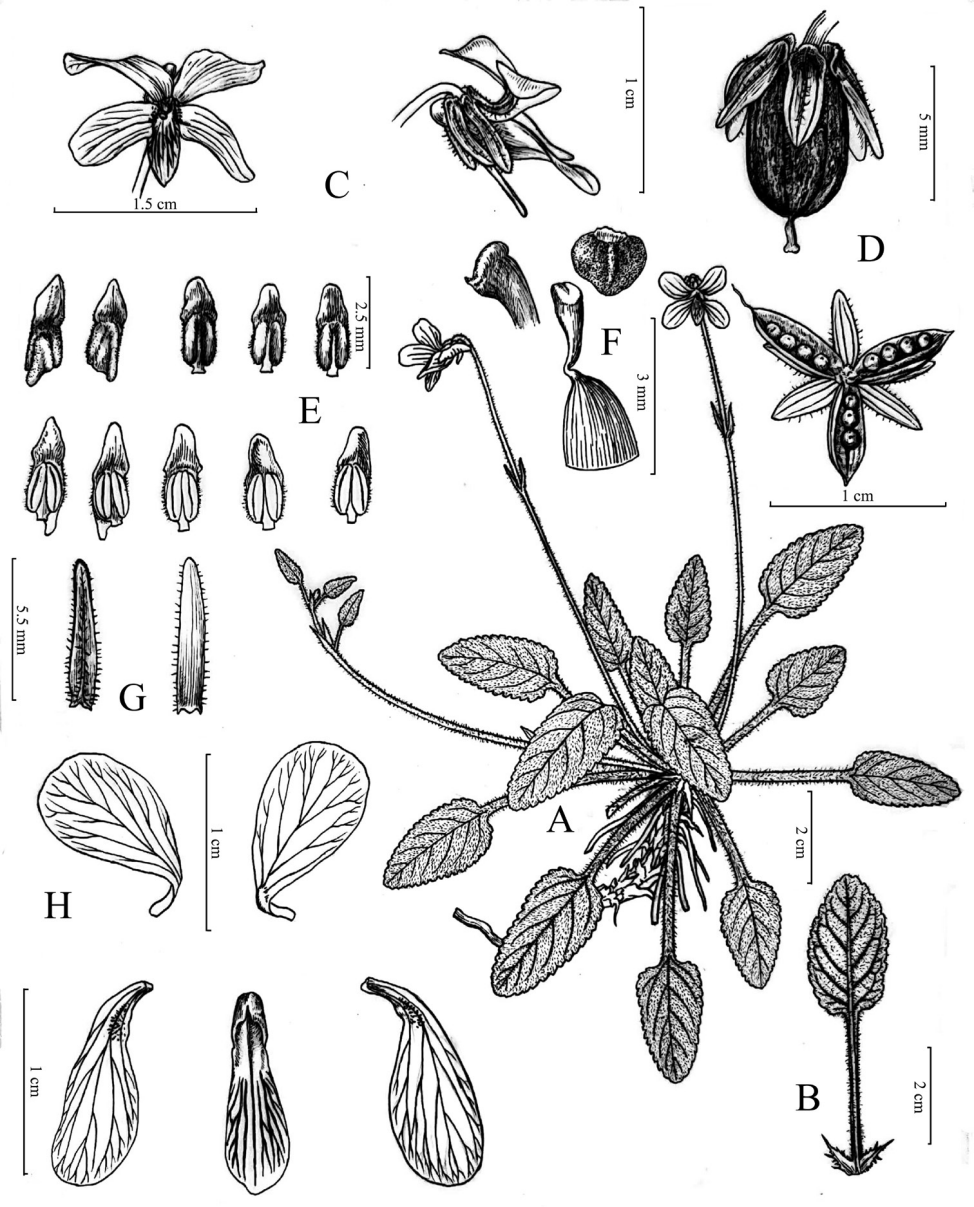
*Viola huizhouensis***A** habit and flowering branch **B** leaf and stipules **C** flower, front view and lateral view **D** young capsule with sepals and bracteoles and dehiscent capsule with seeds **E** stamens in adaxial view (below) and abaxial view (above) **F** stigma and pistil **G** bracteoles **H** petals.

China. Guangdong: Huizhou City, Xiangtoushan National Nature Reserve, Darenyan, 23°15.99'N, 114°22.27'E, 535 m a.s.l., 29 March 2018, *Y. S. Huang* and *Q. Fan 1803* (holotype: SYS; isotypes: IBSC, SYS). (Figs 2, 3)

#### Diagnosis.

*Viola huizhouensis* is most similar to *V. guangzhouensis*, but differs by its much stouter rhizome, lack of aerial stem, different leaf shape and dense pubescence of the basal pedicel and the whole plant.

#### Description.

Herbs, perennial, basal leaves rosulate, 10–15 cm tall. Rhizome erect or obliquely erect, rather stout, 4–7 mm diam.; stolons with an apical rosette of leaves, usually producing adventitious roots. Leaves alternate; stipules leaf-like, base adnate to the petiole, densely pubescent, lanceolate, 6–8 × 1–1.5 mm, apex acuminate, margins sparsely fimbriate or fimbriate-laciniate; petioles densely pubescent, 3–5 cm long, narrowly decurrent-alate; blades narrowly ovate to ovate, apex obtuse, 1.5–3 × 1–2 cm, thinly leathery or chartaceous, densely pubescent, abaxially dark purple, 5 to 7 veins on each side of mid-rib, margin coarsely serrate, base cuneate. Flowers 15–18 mm diam.; pedicels slender, 6–10 cm long, pubescent, usually exceeding leaves, with two opposite bracteoles above middle; bracteoles lanceolate, pubescent, 4–8 mm long, margin entire, apex obtuse. Sepals green, pubescent, linear-lanceolate, 2.7–3.7 × 0.5–1 mm, margin entire, apex obtuse, base truncate or rounded. Petals whitish to light purple, with apparent violet lines, anterior one with a yellow to green patch at base; upper petals, oblong to linear-lanceolate, 2.5–3 × 0.5–0.8 mm, glabrous, margin entire, apex obtuse or erose; lateral petals with glandular hairs at the base adaxially, oblong, 4.5–5 × ca. 1.5 mm, margin entire, apex obtuse or erose; anterior petal with a short saccate spur at base, broadly spathulate or flabellate, margin entire to slightly undulate, apex obtuse. Stamens 5, unequal, puberulent, the anther thecae ca. 1 mm long, terminal appendages ca. 0.7 mm long, the posterior appendages (nectar spurs) of two anterior stamens 0.7–1 mm long. Ovary ovoid to ellipsoid, ca. 0.7 mm diam., puberulent; style ca. 1.0 mm long, conspicuous geniculate at base; stigma thickly margined on lateral sides, slightly raised at central part, shortly beaked at the apex. Capsule with brownish lines at maturity, ovoid, 6–8 mm long. Seeds brown, ovoid, 1–1.5 mm long.

#### Phenology.

Flowering from March to June, fruiting from April to July.

#### Distribution, ecology and conservation status.

Populations of *Viola huizhouensis* were only discovered in Darenyan, Xiangtoushan National Nature Reserve, Guangdong Province. The species was observed to grow on damp cliffs and rocks in broad-leaved forests at altitudes between 400 and 800 m. Its known localities are well protected and more field investigations are needed to determine its distribution.

#### Note.

Based on its slightly 2-lobed stigma and stolons topped by rosettes of leaves, *Viola huizhouensis* should be a member of section Diffusae (W. Beck.) C.J. Wang, which was formerly treated as subsection Diffusae under section Viola by Becker (1925). The closest relative of *V. huizhouensis* on morphological grounds could be *V. guangzhouensis*. They shared several characteristics, for example, the well-developed rhizome and the bearded lateral petals. The new species can be distinguished from *V. guangzhouensis*, however, by its much stouter rhizome; lack of aerial stem; different leaf shape (apex obtuse, never acute vs. apex acute); and dense pubescence of the basal pedicel and the whole plant (vs. the basal pedicel sparsely puberulous or subglabrous and the stem glabrous) (Tables 1, 2; Fig. 2).

The ITS tree shows that *V. huizhouensis* is sister to *V. guangzhouensis* (BS = 100%), then they form a well-supported clade with *V. yunnanensis*, *V. diffusa*, *V. nanlingensis* and *V. lucens* (BS = 99%) (Fig. 1). *Viola guangzhouensis* and the other four species in this clade all belong to section Diffusae (Dong et al. 2009). Thus, the phylogenetic analysis supports *V. huizhouensis* as being close to *V. guangzhouensis* and belongs to section Diffusae.

In conclusion, the morphological differences and the molecular phylogenetic results provide sufficient evidence for treating *V. huizhouensis* as a distinct new species and it is a member of section Diffusae (W. Beck.) C.J. Wang (Wang 1991).

**Table 1.** Morphological differences between the species *V. huizhouensis* and *V. guangzhouensis*.

Characters	*V. huizhouensis*	*V. guangzhouensis*
Leaf shape	narrowly ovate to ovate, apex obtuse, never acute	ovate-triangular to narrowly triangular, apex acute
Leaf margin	coarsely serrate	obtusely dentate
Rhizome	rhizome erect or obliquely erect, rather stout, 4–7 mm diam.	rhizome obliquely ascending, slender, 1–2 mm diam.
Aerial stem	lack of aerial stem	slender, 1–1.5 mm diam., 10–25 cm tall
Pedicel	pedicel basal, 6–10 cm long, densely pubescent	pedicel basal or axillary, 5–8 cm long, sparsely puberulous or subglabrous

**Table 2.** Quantitative characteristics and significant difference analysis of the species *V. huizhouensis* and *V. guangzhouensis*.

Quantitative characteristics	*V. huizhouensis*	*V. guangzhouensis*
l_p_ (mm)	24.4 ± 9.9	53.4 ± 14.9
*l*_m_ (mm)	22.2 ± 5.0	31.4 ± 7.1
*L* (mm)	22.5 ± 5.2	34.8 ± 7.5
*L/ l_m_*	1.01 ± 0.02	1.11 ± 0.04
*N*	304.1 ± 73.9	92.3 ± 16.5
*D* (mm)	4.53 ± 1.47	1.61 ± 0.34

Note: *l_p_* = length of petiole; *l_m_* = distance from the proximal end of the mid-vein to the distal end; *L* = lamina length; *N* = number of the pubescence per 25 mm^2^; *D* = rhizome diameter. Independent-Sample Mann-Whitney Test was used and seven rhizomes and 30 basal leaves were measured for each species, all quantitative characteristics representing significant difference at the 0.5% nominal level.

## Supplementary Material

XML Treatment for Viola huizhouensis

## Appendix 1

**Table:** GenBank accessions for phylogenetic analysis.

Taxon	GenBank accessions
*V. yunnanensis*	FJ002915
*V. diffusa*	FJ002917
*V. nanlingensis*	FJ002916
*V. lucens*	FJ002913
*V. verecunda*	AY928283
*V. triangulifolia*	FJ002912
*V. schulzeana*	FJ002907
*V. rockiana*	FJ002906
*V. biflora*	AY928309
*V. delavayi*	FJ002908
*V. orientalis*	AY928271
*V. principis*	FJ002904
*V. mucronulifera*	FJ002910
*V. moupinensis*	FJ002900
*V. kiangsiensis*	FJ002901
*V. davidii*	FJ002902
*V. grandisepala*	FJ002903
*V. prionantha*	JF830901
*V. philippica*	FJ002895
*V. monbeigii*	FJ002894
*V. pekinensis*	FJ002892
*V. dissecta*	DQ787774
*V. hancockii*	FJ002890
*V. mandshurica*	AY928300
*V. inconspicua*	FJ002897
*V. magnifica*	FJ002899
*V. chaerophylloides*	AY928290
*V. stewardiana*	FJ002883
*V. grypoceras*	AY928280
*V. acuminate*	AY928273
*V. rupestris*	FJ002888
*V. shinchikuensis*	FJ002885
*V. serrula*	FJ002887
*V. labradorica*	FJ002889
*V. collina*	EU413938
*V. atroviolacea*	FJ002878
*V. guangzhouensis* 1	FJ002918
*V. guangzhouensis* 2	MW683480
*V. guangzhouensis* 3	MW683479
*V. huizhouensis* 1	MW683486
*V. huizhouensis* 2	MW683485
*V. huizhouensis* 3	MW683484
*V. huizhouensis* 4	MW683483
*V. huizhouensis* 5	MW683482
*V. huizhouensis* 6	MW683481
*Rinorea bengalensis*	FJ002919

## Supplementary materials

Supplementary material 1 Figure S1 Data type molecular data Explanation note Strict consensus tree derived from ITS sequence with Bayesian posterior probabilities in the nodes. https://binary.pensoft.net/file/531568This dataset is made available under the Open Database License (http://opendatacommons.org/licenses/odbl/1.0/). The Open Database License (ODbL) is a license agreement intended to allow users to freely share, modify, and use this Dataset while maintaining this same freedom for others, provided that the original source and author(s) are credited.Yan-Shuang Huang, Ning Kang, Xiang-Jing Zhong, Wen-Bo Liao, Qiang Fan
