# Supplementary figures and images for: A new species of Viola (Violaceae) from Guangdong Province, China
Source: PhytoKeys. 2021 Apr 16;176:67–76. doi: 10.3897/phytokeys.176.65443 (PMC8065024; doi:10.3897/phytokeys.176.65443)

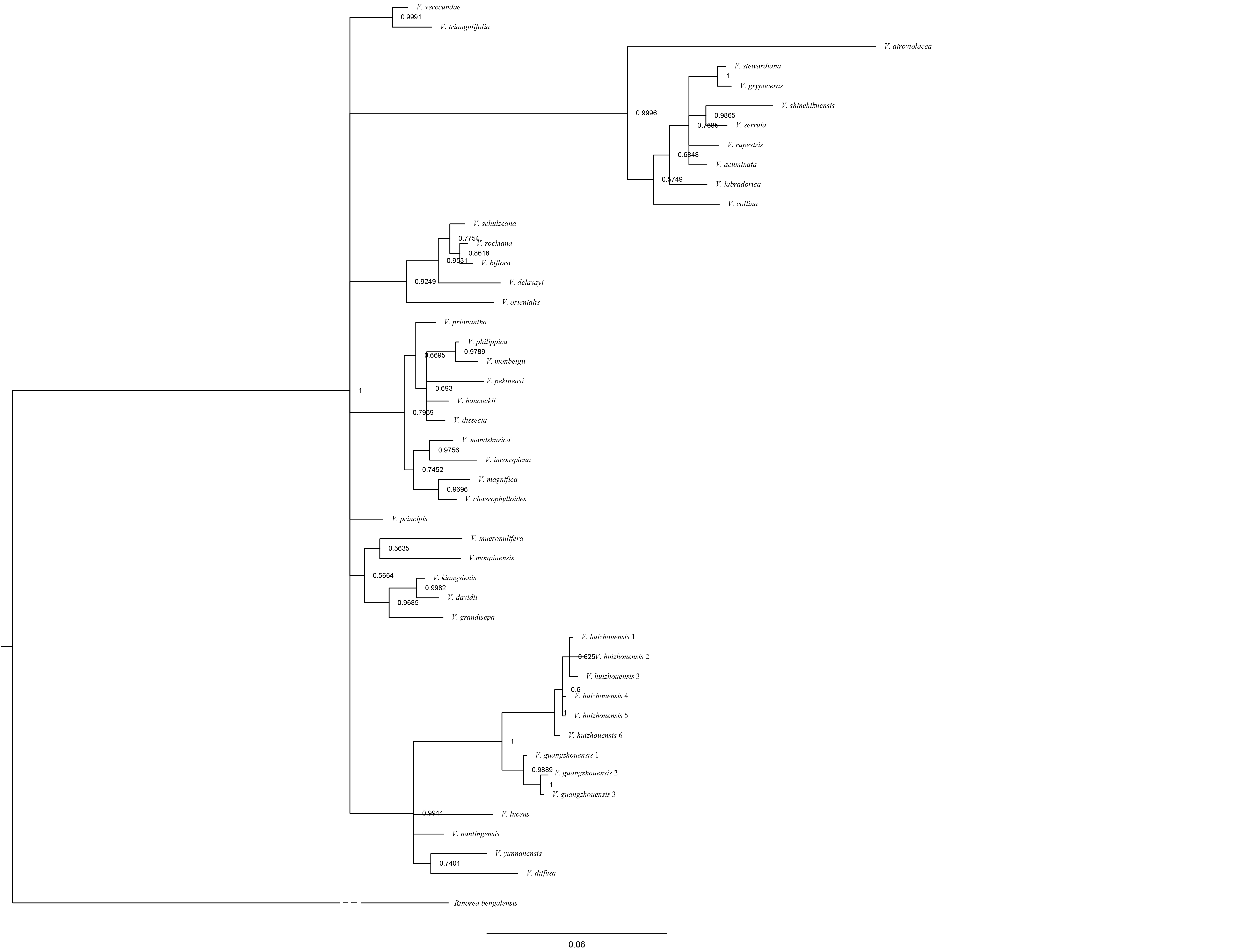

Supplement: Supplementary material 1 — Figure S1 [file phytokeys-176-067-s001.jpg]
